# The Relationship Between Community Size and Iconicity in Sign Languages

**DOI:** 10.1111/cogs.70074

**Published:** 2025-06-07

**Authors:** Shiri Lev‐Ari, Rose Stamp, Connie de Vos, Uiko Yano, Victoria Nyst, Karen Emmorey

**Affiliations:** ^1^ Department of Psychology Royal Holloway, University of London; ^2^ Department of English Literature and Linguistics Bar‐Ilan University; ^3^ Department of Communication and Cognition Tilburg University; ^4^ Anthropological Studies The Graduate University for Advanced Studies, SOKENDAI; ^5^ Centre for Linguistics, Leiden University; ^6^ School of Speech, Language and Hearing Sciences, San Diego State University

**Keywords:** Iconicity, Community size, Language evolution, Sign language

## Abstract

Communication is harder in larger communities. Past research shows that this leads larger communities to create languages that are easier to learn and use. In particular, previous research suggests that spoken languages that are used by larger communities are more sound symbolic than spoken languages used by smaller communities, presumably, because sound symbolism facilitates language acquisition and use. This study tests whether the same principle extends to sign languages as the role of iconicity in the acquisition and use of sign languages is debated. Furthermore, sign languages are more iconic than spoken languages and are argued to lose their iconicity over time. Therefore, they might not show the same pattern. The paper also tests whether iconicity depends on semantic domain. Participants from five different countries guessed the meaning and rated the iconicity of signs from 11 different sign languages: five languages with >500,000 signers and six languages with <3000 signers. Half of the signs referred to social concepts (e.g., friend, shame) and half referred to nonsocial concepts (e.g., garlic, morning). Nonsocial signs from large sign languages were rated as more iconic than nonsocial signs from small sign languages with no difference between the languages for social signs. Results also suggest that rated iconicity and guessing accuracy are more aligned in signs from large sign languages, potentially because smaller sign languages are more likely to rely on culture‐specific iconicity that is not as easily guessed outside of context. Together, this study shows how community size can influence lexical form and how the effect of such social pressures might depend on semantic domain.

Imagine you live in London and need to describe the latest tube strike or the upcoming municipal elections to someone who lives in a village in Scotland. Now imagine that you need to describe the same information to someone in your neighborhood that you interact with regularly. The latter is likely to be easier as you can draw on shared knowledge (e.g., tube stations and schedule, electoral candidates, the electoral system) and on past interactions. When interacting with someone from your neighborhood, you can also be more certain that your pronunciation and lexical choices will be understood, while differences in pronunciation and lexical choice might hinder communication with someone who is from a very different and far‐away location (Chambers & Trudgill, 1998). Interactions with strangers with diverse backgrounds are quite common for members of large language communities, such as English, or American Sign Language. Such interactions, however, are often absent in language communities that are small, especially if they are close‐knit, such that every language user knows almost all other language users. This study investigates whether sign languages with more users have more iconic signs in order to overcome the greater communicative challenges that their users encounter.

We start by reviewing past literature that shows that larger communities create languages that are easier to learn and use in order to overcome the greater communicative challenges they encounter. Next, we discuss whether the reviewed results are likely to extend to the case of sign languages before describing the current study which tested this question.

## 1 Larger communities create more robust languages

In this paper, we define language community as all the users of a language independent of their geographic location. Such language communities encounter more communicative challenges the larger they are. For example, in larger communities, it takes longer for information to travel and there is a greater likelihood that the information will fail to reach all members, which hinders alignment between members. As each individual has their own unique manner of communication, larger communities may also exhibit greater overall variation, all else being equal.^1^ Input variability is an obstacle not only to alignment but is also known to pose a challenge to language learning and language use. For example, individuals show poorer word identification when exposed to input from multiple speakers rather than a single speaker (e.g., Heald & Nusbaum, 2014; Pisoni, 1993), and learning a new phonological contrast from variable rather than more homogeneous input is initially harder and might be less successful for learners with lower perceptual aptitude (e.g., Giannakopoulou, Brown, Clayards, & Wonnacott, 2017; Sadakata & McQueen, 2014). Lastly, in many cases, members of larger communities are more likely to interact with strangers, including ones that come to the interaction with different knowledge and expectations (e.g., Wray & Grace, 2007), which also renders communication more challenging.

Nevertheless, despite the greater challenges encountered by members of larger communities, these speakers communicate successfully. The Linguistic Niche Hypothesis proposes that the reason that members of larger communities communicate as successfully as those from smaller communities is because languages adapt to the communicative needs of the community (Dale & Lupyan, 2012; Lupyan & Dale, 2010). In particular, larger communities seem to develop more robust languages, that is, languages that are easier to learn, process, and use, than smaller communities. For example, languages spoken by more people have less complex morphology and greater systematicity which facilitates learning and comprehension (Koplenig, 2019; Lupyan & Dale, 2010; Raviv, Meyer, & Lev‐Ari, 2019; Reali, Chater, & Christiansen, 2018 but see Koplenig & Wolfer, 2023; Shcherbakova et al., 2023). Larger communities are also more likely to rely on Subject‐Verb‐Object constituent order than smaller communities when delivering potentially confusable information, as this word order assists addressees in disambiguation (Lev‐Ari, 2023).

Of particular interest is a recent study that examined whether languages spoken by larger populations are more sound symbolic, that is, whether the sounds of words in languages with many speakers are more likely to have a nonarbitrary association with the meaning (Lev‐Ari et al., 2021). Sound symbolism is known to facilitate language acquisition in both children (e.g., Imai, Kita, Nagumo, & Okada, 2008; Kantartzis, Imai, & Kita, 2011) and adults (e.g., Nielsen & Rendall, 2012). Sound symbolism has also been shown to facilitate processing, at least under certain circumstances (e.g., Meteyard et al., 2015; Sidhu, Vigliocco, & Pexman, 2020). Additionally, when interacting with strangers, an iconic association between a word and its meaning can help the interlocutor recover the meaning in context if the word is unfamiliar or there is potential ambiguity. In line with this argument, some research suggests that iconicity is more likely to emerge in a communicative context. Specifically, sound symbolism was more likely to emerge in an iterated learning study with generations of pairs playing a communication game than in an iterated study of single users being tested on their memory of the language (Tamariz, Roberts, Martínez, & Santiago, 2018).

Because of the facilitative role of sound symbolism in communication, Lev‐Ari et al. (2021) predicted that communities that encounter greater communicative difficulties would be more likely to rely on sound symbolism in their communication as a tool to overcome their communicative challenges. To test that, the participants listened to synthesized recordings of the translation equivalents of the words *big* and *small* from 20 unfamiliar languages spoken by tens of millions of speakers (e.g., Mandarin, Turkish, Hausa) and 20 unfamiliar languages spoken by only hundreds or thousands of speakers (e.g., Juwal, Talodi, Yele). Participants guessed whether each word meant *big* or *small*. Results indicated that participants were more accurate in guessing the meaning of words from unfamiliar languages spoken by millions of speakers than unfamiliar languages spoken by hundreds or thousands of speakers (Lev‐Ari et al., 2021), supporting the proposal that words in languages with more speakers are more sound symbolic.

## 2 The case of sign languages

The current study examines whether the degree of iconicity in sign languages also depends on community size. On the one hand, research on iconicity shows analogous findings in spoken and signed languages (see Perniss, Thompson, & Vigliocco, 2010, for review), suggesting that the results of Lev‐Ari et al. (2021) should generalize to sign languages. On the other hand, the precise role of iconicity in language acquisition and use is unclear for sign languages (see Ortega, 2017, for review). Early work presented evidence that iconicity had no role in sign language acquisition by deaf children (e.g., Meier & Newport, 1990), but recent studies suggest that more iconic signs are acquired earlier (Caselli & Pyers, 2020; Thompson, Vinson, Woll, & Vigliocco, 2012). Many studies have shown that iconic signs are learned faster and remembered better by hearing second language learners (e.g., Baus, Carreiras, & Emmorey, 2013; Campbell, Martin, & White, 1992; Lieberth & Gamble 1991; Mott, Midgley, Holcomb, & Emmorey, 2020), but there is also some evidence that the phonological form of iconic signs may be retained less accurately, possibly due to interference from the similar but nonidentical form of gestures that convey the same meaning (Ortega & Morgan, 2015a, 2015b). With respect to language use by deaf adults, picture‐naming studies show that iconic signs are retrieved faster than noniconic signs (McGarry, Mott, Midgley, Holcomb, & Emmorey, 2021; Navarrete, Peressotti, Lerose, & Miozzo, 2017; Vinson, Thompson, Skinner, & Vigliocco, 2015); however, recent work suggests that this facilitatory effect on sign production is task‐specific and not found in other paradigms (Gimeno‐Martinez & Baus, 2022; McGarry, Midgley, Holcomb, & Emmorey, 2023; Mott et al., 2020). Thus, the nature of the iconicity advantage for sign language processing and acquisition is still under debate. Nonetheless, theories that explain the emergence of iconicity encompass spoken and signed languages alike (Perlman, Little, Thompson, & Thompson, 2018; Perniss et al., 2010).

Experimental studies of language evolution that employed a visual communication system similarly suggest that the results of Lev‐Ari et al. (2021) should generalize across modalities (Fay & Ellison, 2013; Fay, Garrod, & Roberts, 2008). In these studies, participants interacted via drawings in pairs or groups of eight. Over rounds, the drawings became simpler and more conventional. Crucially, the drawings that were created by the larger groups were better understood by naïve participants than those that were created by the pairs, indicating that larger groups rely more on iconic signals in the visual modality as well.

On the other hand, sign languages are more iconic than spoken languages (Perlman et al., 2018), so signers may not need to increase reliance on iconicity in order to overcome communicative challenges. Furthermore, sign languages have been argued to become less iconic over time as pressures of articulatory ease and systematization lead them to reduce iconic features and become more arbitrary (Frishberg, 1975). Considering that at least the documented sign languages with more users are often older, one might expect them to be less iconic.

Therefore, when considering whether the finding that larger spoken languages are more iconic should generalize to signed languages, there are good theoretical reasons to predict both that it would and that it would not. The study we report evaluates this question while also examining whether the effect of community size depends on the semantic domain. If the greater reliance on iconicity is a response to social pressures, it is possible that the difference between smaller and larger sign languages will be particularly evident in signs referring to social concepts. Larger and more diverse communities might be at risk of lower social cohesion and greater social tension which could make users particularly prone to social misunderstandings compared to other, nonsocial domains, thus motivating more iconic social signs. The study, therefore, compares the iconicity of signs referring to both social and nonsocial concepts from sign languages with large and small communities of users. Before describing the study, however, it is important to consider the relationship between iconicity and transparency (see Sehyr & Emmorey, 2019), and, therefore, the type of iconicity that one should expect to differ between sign languages that serve communities of different sizes.

Spoken language research on sound symbolism tends to distinguish between universal sound symbolic patterns and language‐specific patterns, such as phonesthemes. For example, words starting with *gl* in English often refer to light or vision (e.g., glance, glimpse, glow) but there is no inherent link between the sounds /gl/ and vision or light, and, therefore, this association, as well as other language‐specific systematic patterns are absent in most languages of the world (e.g., Dingemanse, Blasi, Lupyan, Christiansen, & Monaghan, 2015). In contrast, the sound symbolic association between high front vowels, such as /i/, and small size is based on physical properties, such as the higher frequency at which small versus larger objects resonate (Sidhu & Pexman, 2018a) or the (imperfect) association between an animal's size, the size of its vocal chords, and consequently the pitch of the sounds it produces (Ohala, Hinton, & Nichols, 1997). Because the association between vowels with high formant frequencies and small size is not dependent on linguistic knowledge, it is evident as early as infancy (e.g., Peña, Mehler, & Nespor, 2011), and across the world's languages (Blasi, Wichmann, Hammarström, Stadler, & Christiansen, 2016). Correspondingly, research on sound symbolism often asks participants to learn or guess the meaning of words in other languages (e.g., Lockwood, Dingemanse, & Hagoort, 2016; Shinohara & Kawahara, 2010) or examines cross‐linguistic patterns (Blasi et al., 2016).

Iconicity in sign languages is described as a subjective impression that depends partially on the perceiver's knowledge of the language and culture (Occhino, Anible, Wilkinson, & Morford, 2017; Sehyr & Emmorey, 2019). Indeed, when individuals rate the iconicity of signs, signers of that language provide higher iconicity ratings than signers of a different sign language (Occhino et al., 2017), and nonsigners rate some signs (e.g., verbs) as more iconic than users of the language do (Sehyr & Emmorey, 2019). Iconicity (form‐meaning similarity assessed by ratings) is often contrasted with transparency, which is defined as the ability to guess the meaning of a sign out of context (Occhino et al., 2017; Sehyr & Emmorey, 2019). Guessing the meanings of signs presented in isolation is quite challenging—nonsigners correctly guess the meanings of only ∼10% of signs (Bellugi & Klima, 1976; Pizzuto & Volterra, 2000; Sehyr & Emmorey, 2019). No spoken language study (to our knowledge) has asked participants to guess the meanings of sound symbolic words without presenting possible meanings to choose from.

The approach we take in this study is closer to the one employed in research on sound symbolism. In our tasks, we asked nonsigners to make judgments regarding signs from different sign languages. Crucially, our participants were not familiar with the tested sign languages (nor with any other sign language) and resided in different countries than the ones where the sign languages are used, reducing cultural overlap. Additionally, we recruited several groups of participants differing in native language and country of residence, further minimizing any potential cultural links between participants and stimuli. Therefore, the iconicity that we examine is neither language‐ nor culture‐specific.

## 3 The current study

The goal of the study is to investigate whether sign languages with larger communities of users (from now on, *large sign languages*) have signs with greater iconicity than sign languages with smaller communities of users (from now on, *small sign languages*). Additionally, the study explores whether the effect of community size on iconicity depends on semantic domain, and, in particular, is greater for signs denoting social than nonsocial concepts.

To test these questions, participants in this study guessed the meaning (given choices) and rated the iconicity of signs from large sign languages (e.g., Russian Sign Language, Chinese Sign Language) and small sign languages (e.g., Icelandic Sign Language, Kata Kolok—a village sign language in Indonesia). We predicted that the greater communicative challenges that larger communities encounter would lead them to rely more on iconicity. We, therefore, predicted that signs from large sign languages would be rated as more iconic and be more accurately guessed than signs from small sign languages. We additionally predicted that the difference in iconicity and guessing accuracy would be larger for signs referring to social concepts (e.g., friend, to lie) than for signs referring to nonsocial meanings (e.g., rice, morning).

The study was preregistered (AsPredicted.com # 142079).

All materials and results are available on https://osf.io/cvgy2/?view_only=305ff5de700a461681af0265cbd8a5f7

## 4 Method

### 4.1 Participants

We planned on recruiting 180 participants from four different locations for an online study (45 participants per location). Since we replaced two participants as they failed the attention checks in the Guessing task (see below), the final number of participants was 182. The first batch of participants (*N* = 63) was recruited via M‐Turk (www.mtruk.com). They were recruited based on the geographic location of their IP address (France, Germany, or Turkey). As this recruitment method failed to recruit the preregistered number of participants, a second batch of participants (*N* = 119) was recruited via Prolific (www.prolific.com). Participants were recruited based on both geographical location and fluency in the dominant language in that geographic location. As the available number of participants in Turkey was limited, we substituted Turkey as a location with Hungary. We also included a group of participants from Mexico. The final sample consisted of 46 participants from France, 46 participants from Germany,^2^ 45 participants from Mexico, 40 participants from Hungary, and 5 participants from Turkey. Most participants completed the study within 15–20 min. Participants were paid $5/£4 for their participation.

### 4.2 Stimuli

The stimuli consisted of videos of 20 concepts that were each signed in 11 different sign languages by the same deaf model.

Five sign languages with more than half a million users (large sign languages), and six sign languages with fewer than 3000 users (small sign languages) were selected. These languages are commonly used in countries that are different to those from which we recruited our participants. All the large sign languages are deaf community sign languages, that is, languages that emerge when groups of deaf individuals (often from different places) come together, typically for educational purposes when a school for the deaf is established (Meir, Sandler, Padden, & Aronoff, 2010). The small sign languages included both deaf community sign languages and village sign languages,^3^ namely, languages that arise in an existing, relatively insular community, into which a number of deaf children are born (Meir et al., 2010). Table 1 provides information about the sign languages. Both large and small languages in our set had wide geographic distribution. While the geographic distribution of the large and small languages did not perfectly overlap, both sets of languages included one language used in an Eastern European country (Large: Russia, Small: Estonia), and two languages used in South‐East and East Asia (Large: China and India, Small: Japan and Indonesia).

**Table 1:** Characteristics of each sign language

Language name	Community size	Language type	Location	Age	Source
Indian Sign Language	6,815,000	Deaf community sign language	India and Pakistan	∼188 years old	Eberhard et al. (2023)
Chinese Sign Language	4,200,000	Deaf community sign language	China	∼136 years old	Eberhard et al. (2023)
Russian Sign Language	909,000	Deaf community sign language	Russia	∼217 years old	Eberhard et al. (2023)
American Sign Language	860,605	Deaf community sign language	USA	∼206 years old	Eberhard et al. (2023)
LIBRAS	630,000	Deaf community sign language	Brazil	∼166 years old	Eberhard et al. (2023)
Icelandic Sign language	1525	Deaf community sign language	Iceland	∼113 years old	Eberhard et al. (2023)
Estonian Sign Language	1500	Deaf community sign language	Estonia	∼157 years old	Eberhard et al. (2023)
Kata Kolok	1250	Village sign language	Bengkala, Indonesia	∼120 years old	de Vos (2012)
Miyakubo	70	Village sign language	Ōshima island, Japan	∼75 years old	Yano and Matsuoka (2018)
Adamorobe Sign Language	Unknown^a^	Village sign language	Adamorobe village, Ghana	∼200 years old	Nyst (2007)
Kufr Qassem Sign Language	50	Village sign language	Kufr Qassem, Israel	∼90 years old	Stamp, Omar‐Hajdawood, and Novogrodsky (2024)

^a^ There are over 30 deaf signers (Nyst, 2007). It is unknown how many hearing signers there are.

Ten social concepts and 10 nonsocial concepts were selected. Concepts were selected according to their availability in the target sign languages, that is, either known to signers of the target language or available on Spreadthesign.org, a lexical database for sign languages. When selecting the concepts, we tried to avoid signs that were initialized^4^ or fingerspelled,^5^ or emblems. The classification of the concepts into social and nonsocial concepts was originally based on intuition. Diveica, Pexman, and Binney (2023), however, provide socialness ratings for several thousand words, including 9 of the 10 social concepts in our stimuli set (all but *misunderstand*) and 8 of the 10 nonsocial concepts (all but *garlic* and *rice*). With the exception of *learn*, the socialness ratings of *all* social concepts are higher than the socialness ratings of *all* nonsocial concepts (Social: *M* = 5.62, range: 4.77−6.68; Nonsocial: *M* = 2.90, range: 1.64−5.41, range without *learn*: 1.64−4.19).^6^ We ensured that the social and nonsocial concepts were matched for part of speech, rated frequency in ASL (4.29 vs. 4.92, *p*>.3; Caselli, Sevcikova Sehyr, Cohen‐Goldberg, & Emmorey, 2017; Sevcikova Sehyr et al., 2021), log frequency in subtlex‐US (3.31 vs. 3.38, *p*>.8; Brysbaert & New, 2009), and concreteness in English (3.04 vs. 3.57, *p*>.1; Brysbaert, Warriner, & Kuperman, 2014). The social signs conveyed the concepts: angry, friend, gossip, grandparent, (to) lie, (to) marry, (to) misunderstand, shame, sweetheart, and (to) yell. The nonsocial signs conveyed the concepts: 100, (to) buy, (to) finish, garlic, (to) learn, morning, rice, salt, spicy, and (to) wait. Additionally, six signs for six concepts served as attention checks. These signs were highly iconic because their meanings could be guessed with little or no context. These signs were (to) DRINK from Icelandic Sign Language, EYE and (to) SMOKE from ASL, and FIVE, (to) SLEEP, and NO from Indian Sign Language. Participants who made two or more errors in guessing the meaning of the attention check signs were excluded from that task. Similarly, participants who gave at least two of the attention check signs an iconicity rating lower than 5 were excluded.

One native signer of Israeli Sign Language was filmed signing the signs for all concepts. The signer was not familiar with any of the target sign languages with the exception of ASL, of which she had limited proficiency. The signer was presented with videos of the signs produced by members of the community, or, in a few cases, experts of the language. The signer was asked to imitate the presented signs while avoiding affective facial expressions and mouthings (mouth movements derived from the surrounding spoken language), but keeping mouth gestures (mouth movements that are part of the phonological form of the sign). Filming all signs by the same signer who was not proficient in any of the target sign languages reduced the risk that stimuli in the different languages would differ in terms of the signer's expressivity, signing speed, or fluency in the language.

There were a few cases (*N* = 7) in which we could not retrieve the sign for a specific concept for a specific sign language, or the concept was fingerspelled in that language. Therefore, the final dataset consisted of 213 target signs and six attention checks. The target signs were divided into four lists, to avoid participant fatigue. Thus, each of the 213 target signs was presented to 45 participants. Each list consisted of 53 or 54 target signs, such that there were 2–3 signs for each concept, with at least one sign per concept from a large sign language and at least one sign per concept from a small sign language. Across concepts, each list included target signs from all sign languages. Additionally, each list included the six attention checks.

In the meaning guessing task, participants guessed the sign's meaning out of four presented options: the target and three distractors. The distractors were always other concepts from the same domain (social, nonsocial). Each trial consisted of three distractors rather than one to reduce the effect of a specific distractor. That is, we wanted to ensure that participants selected a meaning because it fit the sign best and not because it fit the distractor meaning poorly. When there are three distractors, the influence of each is reduced and participants are more likely to select the best matching meaning rather than eliminate the less compatible meaning. To further reduce any effects of specific distractor concepts, we also created for each concept two, rather than one, distractor sets of three signs each. For example, in half of the trials in which “gossip” was the target sign, the distractors were “angry,” “shame,” and “yell.” In the other half of the trials in which “gossip” was the target sign, the distractors were “grandparent,” “misunderstand,” and “shame.” Because of the limited number of concepts, distractor sets sometimes partly overlapped (e.g., “shame” was a distractor of “gossip” in both distractor sets). Each sign was matched with one distractor set, such that roughly half of the signs for the concept from each language type (large vs. small sign language) were matched with the first distractor set, and the rest with the second distractor set.

### 4.3 Procedure

The study was hosted on Gorilla (www.Gorilla.sc). Instructions and concept names all appeared in English. After consenting to participate in the study and providing demographic information, participants were randomly assigned to one of the four lists of stimuli. All participants first performed the meaning guessing task followed by the iconicity rating task.

In the meaning guessing task, participants were informed that they would be presented with signs from different sign languages and their task is to guess the meaning of each sign. The task was self‐paced. Participants saw all sign videos, one by one, in random order. Each video played automatically but participants could replay it if they wished. Below the video, four potential meanings appeared in a row—the target and its distractor set. The location of the target varied across trials such that it appeared roughly equally in all possible locations (i.e., first word, second word, etc.). Participants did not receive feedback about their accuracy after each video, but they received cumulative feedback every 10 trials and at the end of the task. The cumulative feedback indicated how many of their responses were correct but did not indicate which of the responses were correct and which ones were incorrect. The provision of cumulative feedback made the task more engaging without allowing participants to learn from the stimuli or be able to track the frequency of different meanings.

Following the meaning guessing task, participants performed the iconicity rating task. Participants received a detailed explanation of what iconicity means (e.g., “signs that look like what they mean”), including examples of a highly iconic sign (PUSH in Spanish Sign Language), a noniconic sign (BROTHER in ASL), and an intermediately iconic sign (HIGH in Polish Sign Language). Participants were then asked to rate the iconicity of the signs that they saw in the previous task. Signs were presented with their meaning one by one in random order. The task was self‐paced. The videos played automatically but participants could replay them if they wished. The translation of each sign into English appeared below each video. At the bottom, there was a scale ranging from 1 (Not iconic at all) to 7 (Extremely iconic).

## 5 Results

All analyses were carried out in R 4.0.2 (R Core Team, 2020). All visualizations were generated with the TidyVerse package (Wickham et al., 2019). We removed from all analyses four participants who reported being fluent in at least one sign language.

### 5.1 Iconicity ratings

Before analyzing the data, we examined all responses to ensure that participants did not meet our preregistered exclusion criteria. Five participants met these criteria and were excluded because they gave low iconicity ratings to at least two of the highly iconic attention check signs. The analysis was, therefore, conducted over the remaining 173 participants.

**Fig. 1:**
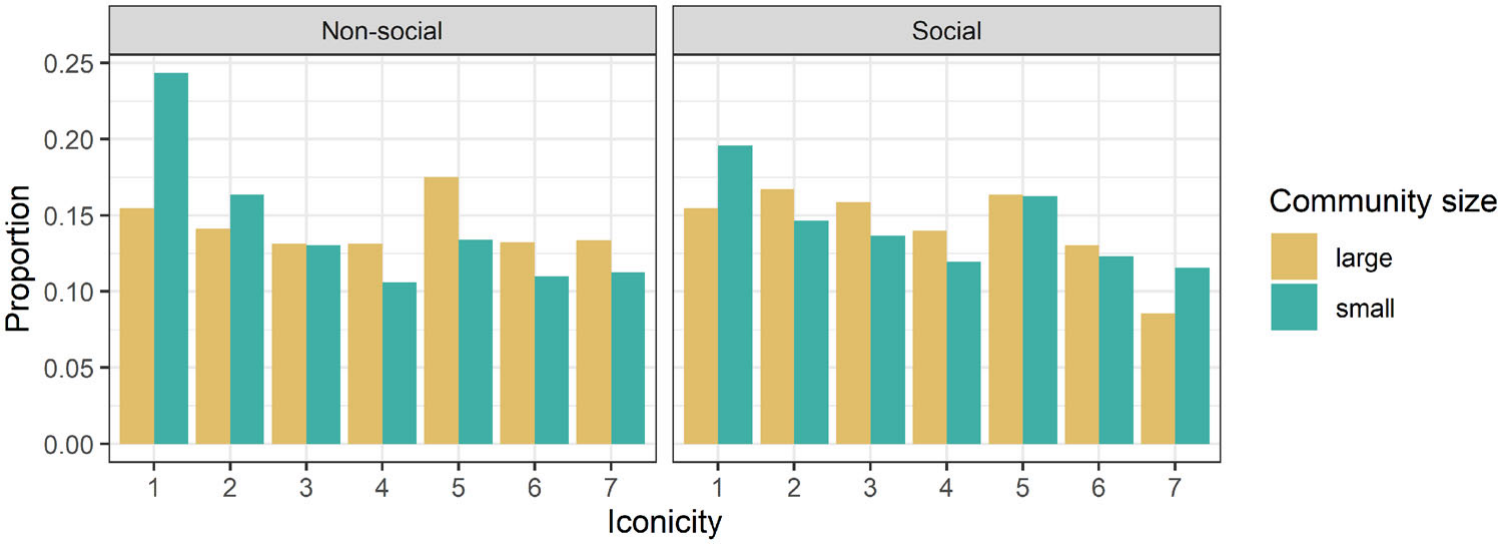
Proportion of each iconicity rating per Community Size (indicated by color) and Semantic Domain (left panel: nonsocial signs, right panel: social signs).

As the dependent measure, iconicity rating, is ordinal, responses were analyzed with a cumulative regression with mixed effects, as implemented with clmm in the ordinal package (Christensen, 2023). The model included Community Size (Large, Small; reference level = Large), Domain (Nonsocial, Social; reference level = Nonsocial), and their interaction as fixed effects, and Participants, and Concepts as random effects. The analysis revealed an effect of Community Size at the reference level (Nonsocial concepts; *β* = −0.43, *SE* = 0.05, *z* = −8.07, *p* < .001) indicating that signs from small sign languages were rated as less iconic than signs from large sign languages. The results also revealed an interaction between Community Size and Domain (*β* = 0.39, *SE* = 0.07, *z* = 5.20, *p* < .001), revealing that, contrary to our hypothesis, the effect of Community Size was restricted to Nonsocial concepts (see Fig. 1).

**Fig. 2:**
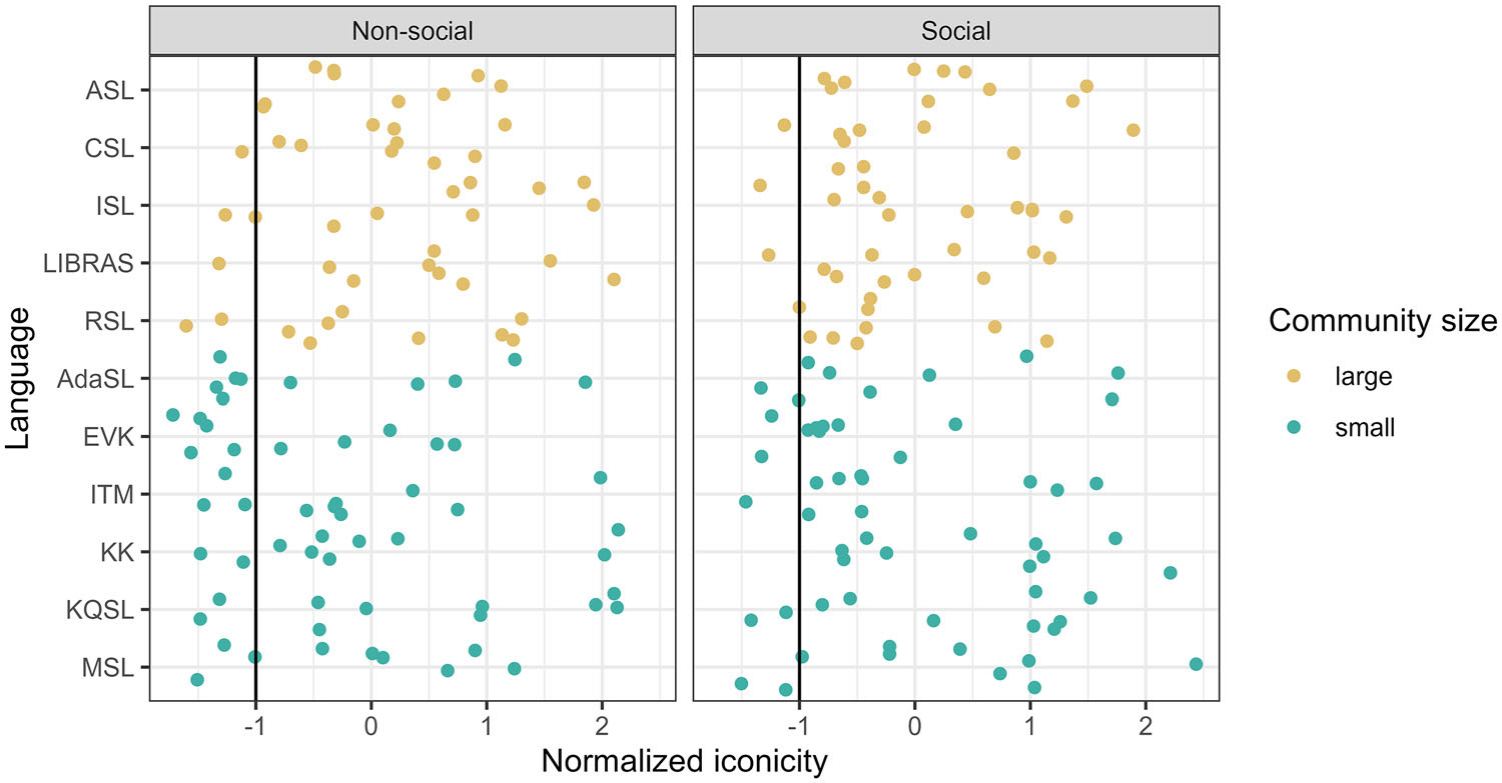
Normalized average iconicity of signs by Language and Semantic Domain. Each dot represents the normalized average iconicity rating for one sign. The y‐axis indicates the language to which the sign belongs with signs from languages with large communities appearing in yellow and signs from languages with small communities appearing in green. The vertical line is placed at 1 SD below the mean. AdaSL = Adamorobe Sign Language; ASL = American Sign Language; CSL = Chinese Sign Language; EVK = Estonian Sign Language; ISL = Indian Sign Language; ITM = Icelandic Sign Language; KK = Kata Kolok; KQSL = Kufr Qasem Sign Language; LIBRAS = Brazilian Sign Language; MSL = Miyakubo Sign Language; RSL = Russian Sign Language.

An examination of Fig. 1 suggests that it is not the case that the distribution of iconicity ratings for signs from large sign languages is shifted to the right, that is, that ratings are similarly distributed but are higher overall. Instead, the difference between the languages is particularly large with regard of ratings of 1, indicating that large sign languages manage to avoid having signs that are particularly low in iconicity. Fig. 2 illustrates the same results but averaged by sign. The figure demonstrates that while signs high in iconicity might be from either large or small sign languages, signs that are particularly low in iconicity, that is, signs that received ratings that are more than 1 Standard Deviation below the mean (left of the vertical line), are almost exclusively from small sign languages (yellow rather than gray dots).

**Fig. 3:**
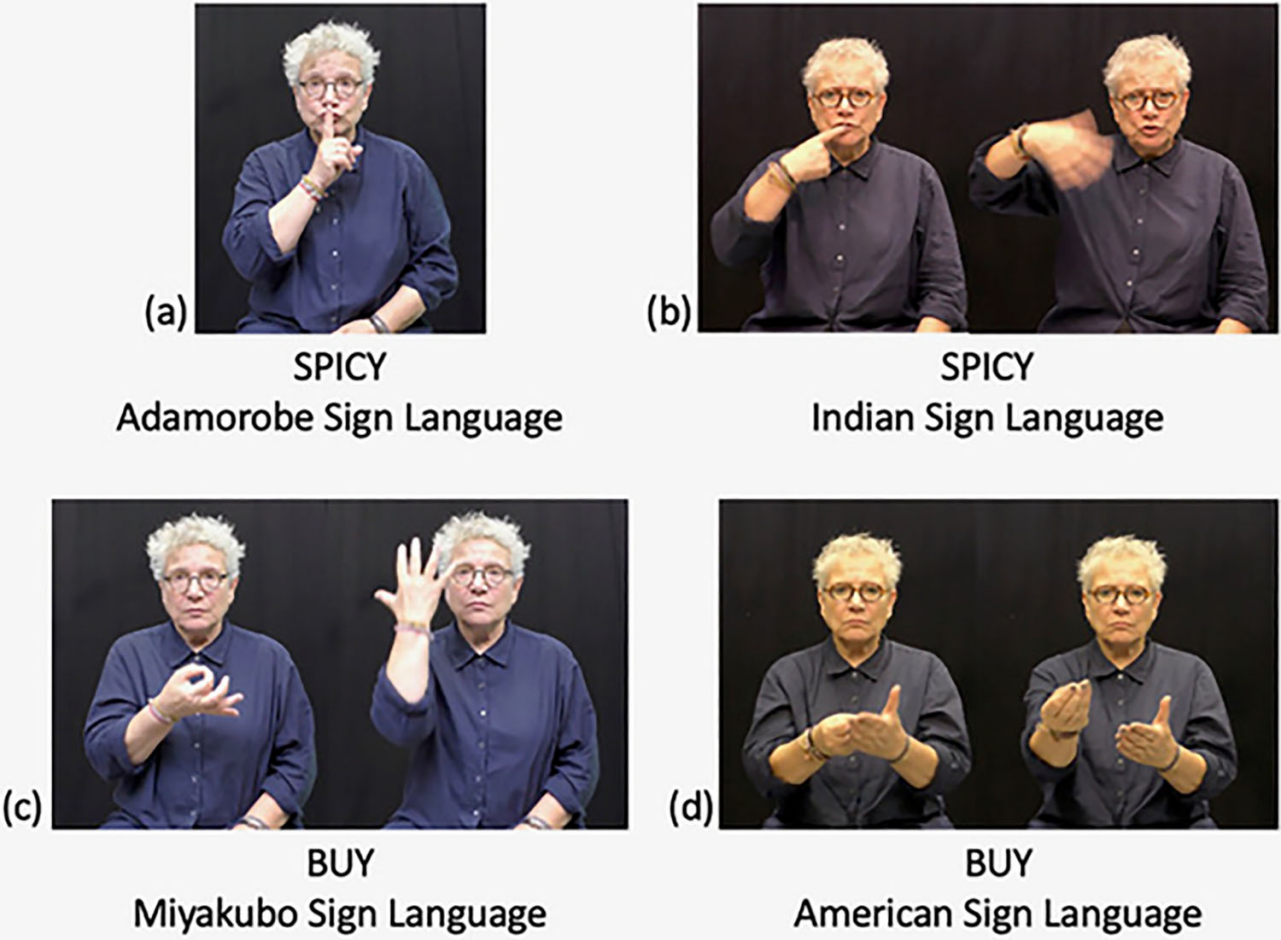
Examples of signs from small and large languages that vary in iconicity: (a) “spicy” in Adamorobe Sign Language (average iconicity rating = 1.67), (b) “spicy” in Indian Sign Language (*M* = 6.32), (c) “buy” in Miyakubo Sign Language (*M* = 1.25), (d) “buy” in ASL (*M* = 5.27).

Fig. 3a‐d provides a few examples of signs particularly low and high in iconicity. Fig. 3a shows the sign for “spicy” in Adamorobe Sign Language, whose average iconicity rating was 1.67 and Fig. 3b shows the sign for “spicy” in Indian Sign Language, whose average iconicity rating was 6.32. Fig. 3c, d shows the Miyakubo and ASL signs for “buy,” respectively, which received average iconicity ratings of 1.25 and 5.27, respectively.

#### 5.1.1 Robustness checks

While both the set of languages and the backgrounds of participants are more varied than in most studies, one may wonder whether the results are robust and generalizable, especially considering the fact that iconicity ratings are culture‐specific (Occhino et al., 2017; Sehyr & Emmorey, 2019). To increase the generalizability of our findings, participants were recruited from different countries than those associated with the languages. Nevertheless, to examine whether the match between participants’ background and the selected languages influenced results, we carried out a follow‐up analysis that tested whether results differed for different participant country–language combinations. That is, we tested whether participants from some countries found the signs from specific languages more or less iconic than participants from other countries. A cumulative regression with iconicity rating as the dependent measure, Participant's Country, Tested Language, and their interaction as fixed effects, and Concept and Participant as random variables revealed several significant interactions. Participants from Germany gave significantly lower iconicity ratings to signs from Icelandic Sign Language (*β* = −0.58, *SE* = 0.25, *z* = −2.30, *p* = .02), Russian Sign Language (*β* = −0.56, *SE* = 0.25, *z* = −2.22, *p* = .03), and Indian Sign Language (*β* = −0.51, *SE* = 0.25, *z* = −2.02, *p* = .04) than participants from other countries. In contrast, participants from Turkey gave higher iconicity ratings to Indian Sign Language (*β* = 1.17, *SE* = 0.59, *SE* = 1.99, *p* = .046) than participants from other countries. Considering Germany is a Western European country that was previously partly under soviet rule, one might expect the sign languages of Iceland and Russia to be the closest to it culturally, leading to higher, rather than lower, iconicity ratings. In other words, the effects that were found seem to go in the opposite direction than the one that would be predicted by cultural specificity. It is less clear why German participants gave lower ratings to signs from Indian Sign Language (except that they seem to give low ratings in general) or why Turkish participants gave higher iconicity ratings to signs from Indian Sign Language. Importantly, Icelandic Sign Language was categorized as a small language, whereas Russian Sign Language was categorized as a large language. Therefore, the pattern that German participants exhibit does not bias the results in favor or against our hypothesis. Additionally, signs from Indian Sign Language were rated both higher and lower than average by different groups, removing any systematic bias toward or against large languages. We, therefore, do not find any evidence that our results were influenced by the proximity between participants’ cultural background and the culture in which the languages are used. Further, the interactions we found should not systematically bias our results.

Despite the similarity in the performance of participants from different countries, one may wonder whether this is because of the similarity in participants’ background. While Hungary, France, Germany, and Turkey are culturally distinct, they are all European countries, so participants from these countries might respond similarly. Additionally, these participants may all be familiar with American culture, conferring an advantage to ASL. Furthermore, while Mexico is not a European country, its proximity to the US and Brazil might confer an advantage when processing and rating signs from ASL and LIBRAS. To address this potential advantage of some of the larger languages in our dataset, we conducted additional analyses focusing on only a subset of the languages, such that small and large languages in this subset shared a similar geographical origin, and none were from the Americas or Western Europe. Specifically, our analysis included responses to two Eastern European languages—one large and one small (Russian Sign Language and Estonian Sign Language), and four East and South‐East Asian languages—two large and two small (Chinese Sign Language, Indian Sign Language, Kata Kolok, and Miyakubo). Results of this analysis reveal the same effects as for the analysis over the entire dataset: Signs from larger sign languages were rated as more iconic than signs from smaller sign languages (*β* = −0.67, *SE* = 0.07, *z* = −9.23) at the reference level (nonsocial signs), and this effect interacted with Domain (*β* = 0.61, *SE* = 0.10, *z* = 6.00). It seems then that the higher iconicity ratings of nonsocial signs from larger languages are not due to the greater cultural affinity between our participants and the included larger languages.

### 5.2 Guessing accuracy

First, we examined whether any participants failed the preregistered attention checks. Two participants incorrectly guessed the meaning of at least two of the six attention check signs and were, therefore, excluded. The analyses were, therefore, conducted on the remaining 176 participants.

**Fig. 4:**
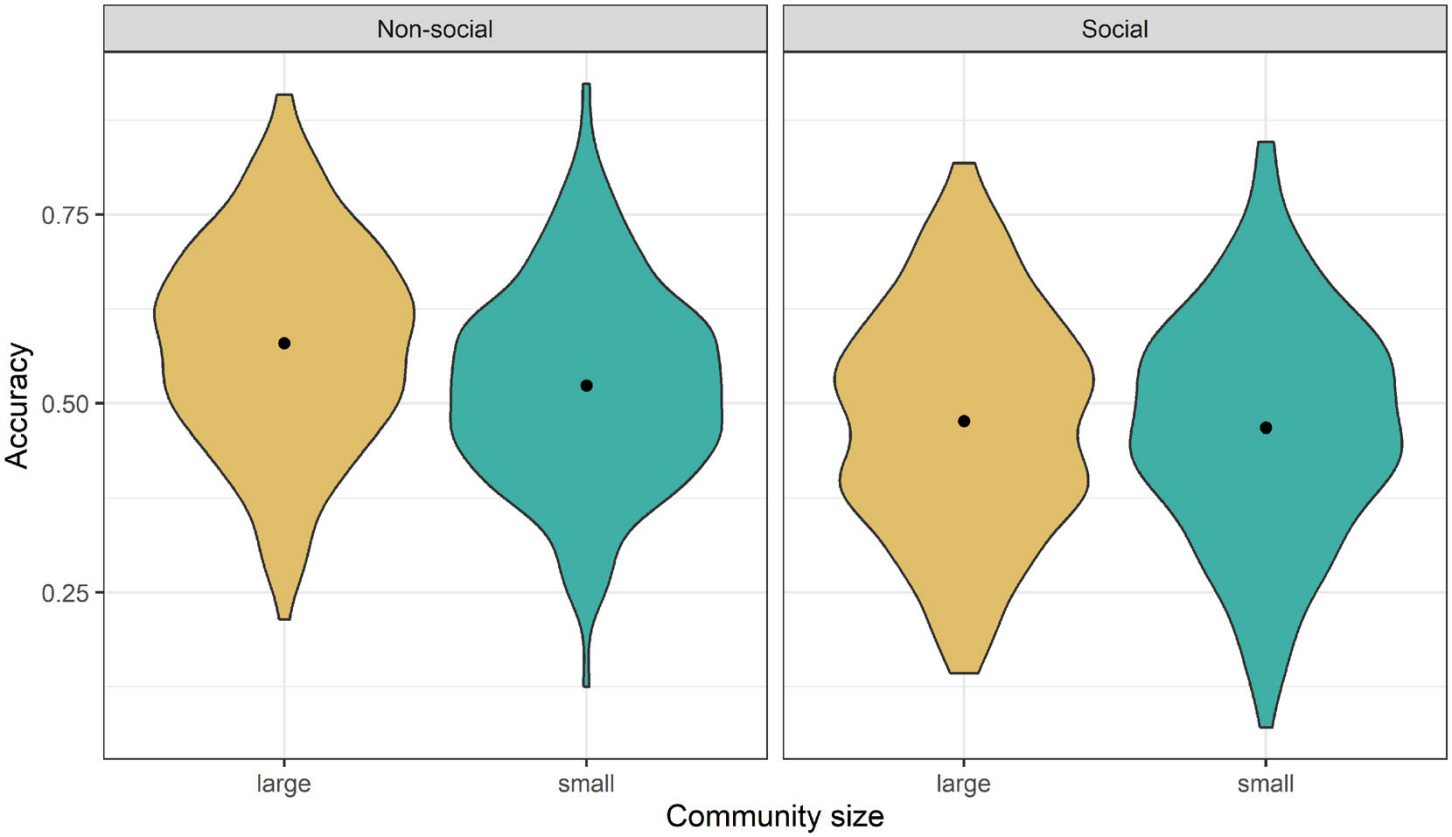
Guessing accuracy as dependent on Community Size and Semantic Domain. The figure plots the distribution of the average accuracy of each individual in each condition. The left panel shows the accuracy of signs denoting Nonsocial concepts and the right panel shows the accuracy of signs denoting Social concepts. Black dots indicate condition averages.

We analyzed accuracy with a logistic mixed effects regression model with the package lme4 (Bates, Maechler, Bolker, & Walker, 2016). The predictors were Community Size (Large, Small; reference level = Large), Domain (Nonsocial, Social; reference level = Nonsocial), Distractor Similarity, and the interaction of Community Size and Domain as fixed effects. Distractor Similarity was included to control for differences in trial difficulty due to differences in semantic similarity between the target and the distractors. Distractor similarity was coded as the cosine similarity between the target and the most similar distractor, based on the genism package (Rehurek & Sojka, 2011) in Python and the built‐in corpus text8.^7^ Cosine similarity can range from −1 to 1. In our set, it ranged from 0.02 (similarity between “lie” and “angry”) to 0.84 (similarity between “spicy” and “garlic”). The statistical model also included intercepts for Participants and Concepts as random variables, a by‐participant slope for Domain, and a by‐concept slope for Community Size. The model did not include a by‐participant slope for Domain as that led the model to fail to converge. While numerically, the pattern of results was similar to that of the analysis of Iconicity, namely, higher numeric accuracy for nonsocial signs from Large sign languages than for nonsocial signs from Small sign languages with no difference for Social signs, these effects were not significant (see Fig. 4). The only significant effect was of Distractor Similarity (*β* = −1.72, *SE* = 0.39, *z* = −4.45, *p* < .001) reflecting the fact that accuracy was higher when the distractors were semantically less similar to the target. While we can only hypothesize why the model did not yield significant effects, it is worth noting that logistic regressions tend to require larger samples than linear regressions to detect comparable effect sizes.

**Fig. 5:**
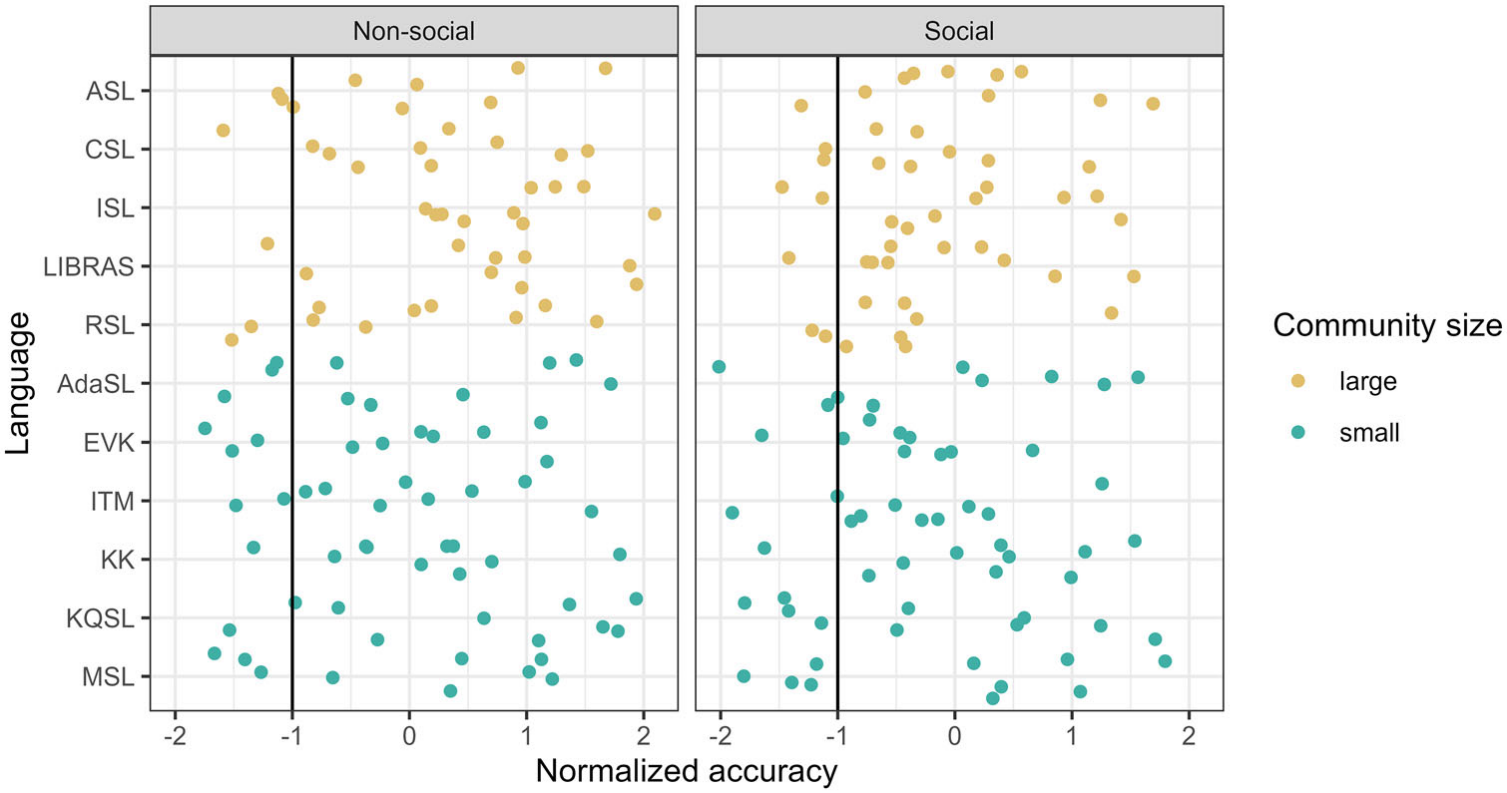
Normalized guessing accuracy of signs by Language and semantic Domain. Each dot represents the normalized average guessing accuracy of one sign. The y‐axis indicates the language to which the sign belongs, with signs from languages with large communities appearing in yellow and signs from languages with small communities appearing in green. The vertical line is placed 1 SD below the mean. AdaSL = Adamorobe Sign Language; ASL = American Sign Language; CSL = Chinese Sign Language; EVK = Estonian Sign Language; ISL = Indian Sign Language; ITM = Icelandic Sign Language; KK = Kata Kolok; KQSL = Kufr Qasem Sign Language; LIBRAS = Brazilian Sign Language; MSL = Miyakubo Sign Language; RSL = Russian Sign Language.

Similarly, examining the pattern of results shows that, despite the null effect, most of the signs with average guessing accuracy that is more than 1 Standard Deviation below the mean were from Small sign languages (see Fig. 5).

#### 5.2.1 Robustness checks

We tested whether participants’ ability to guess the meaning of the signs depended on participants’ background and language combinations. A logistic mixed effects model with Accuracy as the dependent variable, Participants’ Country, Tested Language, and their interaction as fixed effects, and Concept and Participants as random variables did not reveal any interactions between Tested Language and Participants’ Country (all *p*s>.17).

### 5.3 Relationship between iconicity and accuracy

Our study employed two measures to evaluate the iconicity of signs: iconicity ratings and guessing accuracy (given a choice of four meanings). While the two measures are related, they are not identical. In particular, a person may be able to recover the iconicity of a sign when they know what the sign means yet interpret the iconicity erroneously without that knowledge (see Sehyr & Emmorey, 2019, for examples of this pattern). We wondered whether iconicity and accuracy are more associated in Large sign languages than in Small ones.

To test that, we first normalized the average iconicity ratings of the signs. Then we conducted a logistic mixed effects regression analysis with guessing accuracy as the dependent measure, and Normalized Iconicity, Community Size (Large, Small, reference level = Large), Distractor Similarity, and the interaction between Normalized Iconicity and Community Size as fixed effects. The random structure included intercepts for Participants and Concepts and a by‐participant slope for Distractor Similarity and a by‐concept slope for Community Size. The model did not include a by‐participant slope for Community Size as that led the model to fail to converge. Results revealed that Normalized Iconicity predicted guessing accuracy (*β* = 1.02, *SE* = 0.04, *z* = 22.91, *p* < .001). The model also revealed an (expected) effect of Distractor Similarity (*β* = −1.43, *SE* = 0.41, *z* = −3.52, *p* < .001) such that accuracy was lower when the semantic similarity between the target and the distractors was higher. Community Size did not have an effect and did not interact with Normalized Iconicity. In other words, iconicity predicted accuracy in both types of languages to the same degree.

**Fig. 6:**
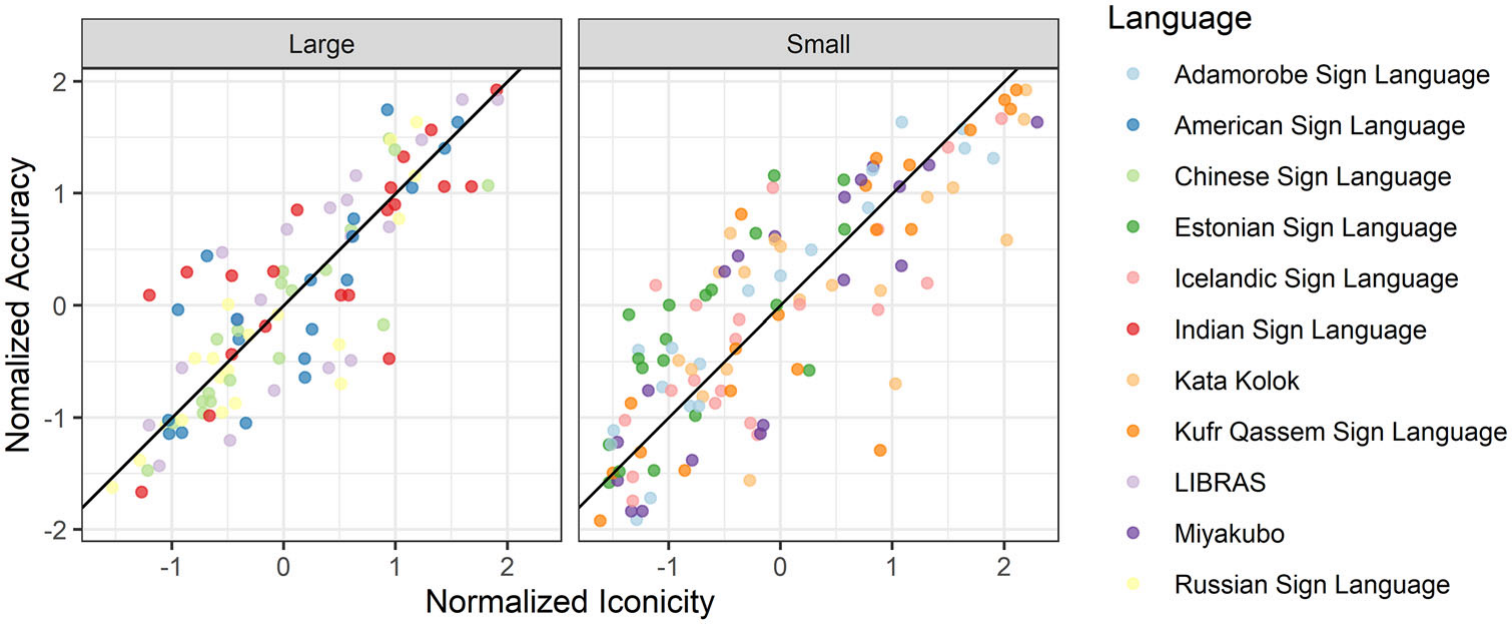
The correlation between Normalized Iconicity Ratings and Normalized Accuracy for signs from Small and Large sign languages. Each dot represents the normalized averages for one sign. Colors indicate the sign language.

At the same time, an examination of the relationship between iconicity ratings and guessing accuracy at the sign level suggested that there are cases where a sign was rated as high in iconicity but was poorly guessed and vice versa. These cases seemed to be more common in Small sign languages than Large ones (see Fig. 6). For example, the sign for “friend” in Kufr Qassem Sign Language was rated as relatively iconic (*M* = 4.95, 0.91 SDs above the mean), yet it was guessed correctly on only 18% of trials (1.30 SDs below the mean, as well as below chance level, 25%). The sign for “friend” in Kata Kolok was similarly rated as high in iconicity (*M* = 5.09, 1.01 SDs above the mean) while being poorly guessed (*M* = 33%, 0.72 SDs below the mean). An exploratory analysis examined whether such discrepancies between iconicity ratings and guessing accuracy were more common in Small sign languages than Large ones. To examine that, we took the normalized average iconicity rating and the normalized average accuracy for each sign. We then calculated the Mismatch Score, namely, the absolute distance between the normalized iconicity rating and the normalized accuracy. For example, the Mismatch Score for the sign “friend” in Kufr Qassem Sign Language is |0.91−(−1.30)| = 2.21. A linear regression with Community Size as a predictor and Mismatch Score as a dependent measure revealed a significant effect of Community Size (*β* = 0.12, *SE* = 0.05, *t* = 2.23). That is, signs from Small sign languages had greater discrepancy scores, indicating that guessing accuracy and rated iconicity are less aligned in Small sign languages than in Large ones.

### 5.4 Concreteness and iconicity

It is often argued that concrete concepts have greater iconic potential than abstract ones (Lupyan & Winter, 2018; Perlman et al., 2018). In fact, it has been argued that one of the reasons that language is not more iconic despite iconicity's facilitative effect on language learning and processing (e.g., Imai et al., 2008; Kantartzis et al., 2011; Meteyard, Stoppard, Snudden, Cappa, & Vigliocco, 2015; Nielsen & Rendall, 2012; Sidhu et al., 2020) is the fact that language also consists of abstract concepts and these do not lend themselves to iconic depiction as well (Lupyan & Winter, 2018). Therefore, we decided to conduct exploratory analyses of the relation between concreteness and iconicity.

**Fig. 7:**
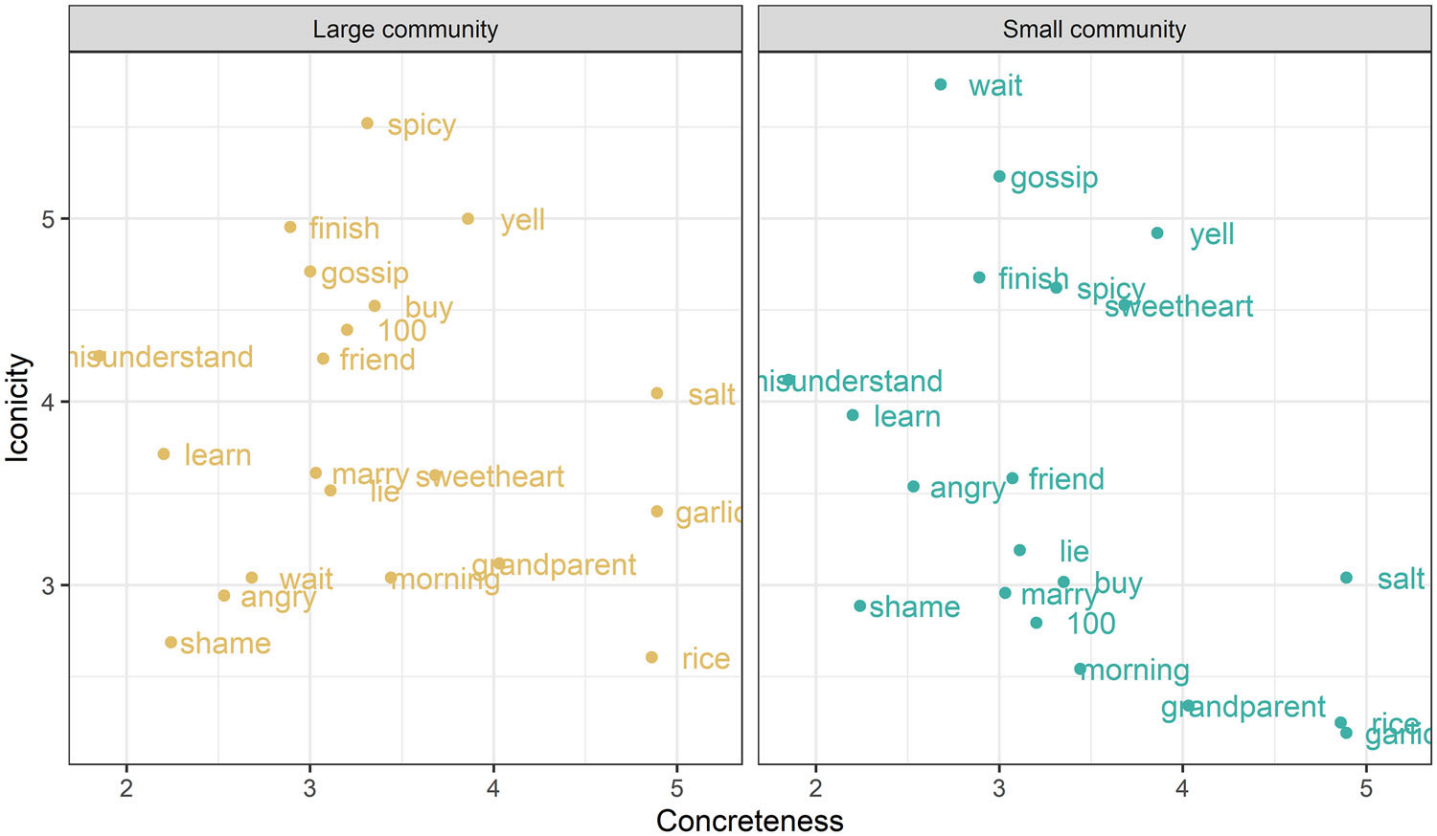
The effect of Concreteness on iconicity ratings for signs from Large (left panel) and Small (right panel) sign languages. Each dot represents the average iconicity rating for signs for the labeled concept across sign languages with the specified community size.

To examine whether iconicity ratings were influenced by the concepts’ concreteness, we conducted a cumulative regression with mixed effects with rated iconicity as the dependent variable and Community Size (Large, Small; reference level = Large), Concreteness (scaled), and their interaction as fixed effects and Participants and Items as random variables. Concreteness values were taken from Brysbaert et al. (2014). The analysis revealed an effect of Concreteness at the reference level (Small communities; *β* = −0.57, *SE* = 0.16, *z* = −3.54, *p* < .001), and an interaction between Concreteness and Community Size (*β* = 0.52, *SE* = 0.24, *t* = 2.14, *p* = .03; see Fig. 7). The interaction reflected the fact that concreteness was *negatively* correlated with rated iconicity in signs from Small sign languages but was not associated with rated iconicity for signs from Large sign languages.

It seems, then, that contrary to prior proposals, abstraction is not an obstacle for iconicity. Curiously, Winter, Lupyan, Perry, Dingemanse, and Perlman (2024) similarly found a negative correlation between iconicity and concreteness in spoken English when using concreteness ratings from Brysbaert et al. (2014), as we did, despite finding a positive correlation between iconicity and concreteness when using alternative measures of concreteness. It is worth noting that the least concrete concepts in our sets were often actions (wait, finish, misunderstand, learn) and actions might lend themselves to iconic depiction better than other word classes (e.g., Perlman et al., 2018; Sehyr & Emmorey, 2019). Furthermore, the study was not set to test the effect of concreteness. Therefore, there was no attempt to sample equally across all levels of concreteness or to control for potential confounds between concreteness and other variables (e.g., frequency, word class).

## 6 Discussion

Communication is harder in larger communities (Dale & Lupyan, 2012). One of the challenges that larger communities face is that larger communities are likely to have greater diversity of backgrounds and, therefore, greater differences in knowledge and expectations than smaller communities. An increase in community size also slows down and limits the spread of information further limiting alignment in communication. Languages spoken by larger communities adapt to these communicative challenges by creating languages that are more robust, that is, easier to learn and use (Dale & Lupyan, 2012; Fay et al., 2008; Lev‐Ari et al., 2021; Lupyan & Dale, 2010; Raviv et al., 2019). One of the adaptations that spoken languages with larger communities display is a greater reliance on sound symbolism (Lev‐Ari et al., 2021). The current study tested whether sign languages with larger communities of users similarly rely on iconic form‐meaning mappings to a greater extent than sign languages of smaller communities. The study also tested whether the effect of community size is particularly evident in signs for social concepts, as community size might disproportionally increase the risk of experiencing greater social misunderstanding than misunderstanding in other domains.

The results of the study demonstrated that community size can indeed influence the iconicity of the signs. At the same time, in contrast to our prediction, results showed that large sign languages rely more on iconicity when communicating about nonsocial concepts, but not when communicating about social concepts. An exploratory analysis suggested that the effect of community size on iconicity stemmed from large sign languages’ avoidance of noniconic signs. That is, all sign languages included many iconic signs, but small sign languages also used many signs low in iconicity, whereas large sign languages avoided them more. The effect of community size on iconicity demonstrates the influence of social pressures on linguistic form. The results support the hypothesis that large sign languages, similarly to large spoken languages, evolve to become easier to learn and use, in order to overcome the greater communicative challenges that they encounter.

The finding that larger sign languages use more iconic signs when communicating about nonsocial concepts is particularly interesting considering that larger languages tend to be older and have larger vocabularies, and iconicity has been argued to diminish over time (Frishberg, 1975) and to be less prevalent as vocabulary increases in size (Brand, Monaghan, & Walker, 2018). That said, while the youngest languages in our dataset were smaller sign languages (Miyakubo—∼75 years old, Kufr Qasem Sign Language—∼90 years old, Icelandic Sign Language—∼113 years old) and the oldest ones were large sign languages (Russian Sign Language—∼217 years old, ASL—∼206 years old), there was quite a bit of overlap in the ages of the languages in the two sets: Small sign languages: 75 years old (Miyakubo)—200 years old (Adamarobe Sign Language); Large sign languages: 136 years old (Chinese Sign Language)—217 years old (Russian Sign Language).^8^

So, why was the effect of community size particularly evident when communicating about nonsocial concepts? One speculative explanation is that it is easier to create a structured mapping between nonsocial concepts and linguistic form. Structure‐mapping models of iconicity posit that selected features of a semantic representation are mapped to the relevant linguistic articulators (Emmorey, 2014; Taub, 2001). It is possible that semantic features of nonsocial concepts are more easily mapped to the visual‐manual phonological form of sign language (e.g., visual shape features for “rice” and “garlic”; digit features for “100”; transfer direction for “buy”). There may be communicative pressure for larger communities to adopt signs with more robust structure mapping. Another possibility for this finding relates to the fact that signs with low iconicity ratings can fall into two groups: signs with arbitrary form‐meaning mappings and signs with culture‐specific iconic mappings that are not easily guessed by outsiders. We hypothesized that the greater diversity among members of larger communities would impose pressure to favor iconic mappings that do not rely on cultural knowledge. It might be the case, however, that social signs are a particularly strong marker of group identity and that there is a wish to monitor and guard entry into the group. Alternatively, language communities might diverge less with regard to social concepts and traditions (e.g., kinship relations) compared to nonsocial ones (e.g., food types), reducing pressure for iconic signs to be guessable. Further research should investigate these possibilities. At the same time, while social and nonsocial concepts were matched for word class, concreteness, and frequency, there might be other differences between the social and nonsocial concepts that we did not consider and lead community size to influence signs in these domains differently.

Our results also revealed that the association between rated iconicity and guessing accuracy is stronger for large sign languages. This finding is in line with our prediction that languages with smaller communities would rely more on culture‐specific or ambiguous iconicity. Such ambiguous iconicity might be recovered when the meaning is provided, leading to high iconicity ratings, but it presents difficulty when guessing with little context. At the same time, our mismatch measure included misalignments between iconicity and guessing accuracy in both directions, that is, it includes not only cases in which a sign was poorly guessed yet rated as iconic but also cases where the meaning of the sign was better guessed than would be predicted by its rated iconicity. The difference between small and large sign languages seems to be due to both types of misalignments, which is different from the expected pattern. We are unsure why this pattern emerged. It is important to keep in mind that guessing accuracy depends not only on the link between the sign and its meaning, but also on the link between the sign and the distractor meanings, which introduces some noise into the measure. Lastly, while we preregistered our prediction regarding a less strong link between guessing accuracy and rated iconicity in small sign languages, the analysis we preregistered was different from the one that shows differences between small and large sign languages, so the results should be treated with caution.

Finally, we examined the relationship between concreteness and iconicity as prior research proposed that it is the need to refer to abstract concepts that limits the prevalence of iconicity (Lupyan & Winter, 2018). According to this proposal, concreteness should correlate with iconicity ratings, and indeed, prior studies revealed such a correlation for ASL and BSL, as well as English, but not Spanish (Perlman et al., 2018; Sidhu & Pexman, 2018b), though one study found that the direction of the correlation between concreteness and iconicity, at least in spoken English, differs for different measures of concreteness (Winter et al., 2024). To our surprise, concreteness was not positively associated with iconicity ratings in sign languages of either type. Instead, we found a negative correlation between concreteness and rated iconicity in sign languages with small communities of users, whereas the two measures were unrelated in large sign languages. This pattern might reflect reliance on culture‐specific iconicity for the concrete concepts in the small sign languages. Signs for grandparents, among the more concrete concepts in our set, often referred to specific coiffures, facial hair, or jewelry, which differ by culture. Correspondingly, these signs often received low iconicity ratings because the iconicity was not understood by our participants. In contrast, the representation of more abstract concepts like “wait” or “finish” might not illustrate subtleties of use but refer to the more general aspects of the concept that are shared cross‐culturally. In other words, it might be the case that concreteness and iconicity are not negatively associated in small sign languages, but that the iconicity of concrete concepts is more likely to be culture‐specific in small sign languages, leading to the negative association between concreteness and rated iconicity. As sign languages with larger communities avoid culture‐specific iconicity (see also Mudd, de Vos, & de Boer, 2022, 9), they do not show an association between rated iconicity and concreteness. It is important to keep in mind though that this was an exploratory analysis and the study was not set to test the relationship between concreteness and iconicity, so concreteness might be confounded with other variables. Nonetheless, the analysis does suggest that the relationship between concreteness and iconicity might depend on the type of iconicity that is examined, that is, form‐meaning mappings that can be recognized by members outside a community or iconic mappings that can be perceived only by members of the community. It will be interesting to examine whether prior reports of a positive correlation between concreteness and iconicity would persist if the raters are not community members or nonsigners from the same geographic location, but members of culturally distant communities.

### 6.1 Potential cultural confounds

Our study included stimuli from 11 languages that span five continents. Our participants were also relatively varied but were all from either Europe or North America. One may, therefore, wonder whether our results generalize to other participant‐language combinations or non‐Western participants, as iconicity ratings are to some extent culture‐specific (Occhino et al., 2017; Sehyr & Emmorey, 2019). Our robustness checks did not reveal any culture‐specificity in participants’ responses. Some of the few differences that were found, lower iconicity ratings for Russian Sign Language and Icelandic Sign Language by German participants, go against the prediction that cultural ties and cultural similarity should increase iconicity ratings. Others do not seem to fit any specific cultural affinity (Turkish and German participants rating signs from Indian Sign Language higher and lower than other groups, respectively). Furthermore, participants rated nonsocial signs from larger sign languages as more iconic than those from smaller sign languages even when the subsets of small and large languages were matched in terms of geographic origin and did not include sign languages from Western Europe or the Americas. This further reduces the likelihood that the higher iconicity ratings for languages from large sign languages are driven by cultural affinity. Nevertheless, future research should test whether participants from non‐Western countries exhibit the same bias.

Future research could also take an experimental approach and examine the evolution of iconicity with an iterated language experiment with groups of different sizes. The signs that the large versus small groups create can then be presented to naïve participants. Based on our study, we would predict that the signs from the larger groups will be rated as more iconic. Such a study would also provide evidence for the causality of the effect of community size on iconicity. Note though that while such a result would not be driven by culture‐language match, this type of study would be less likely to reveal community size differences that are driven by generating signs that rely on culture‐specific versus more general iconicity (as all participants will belong to the same culture).

One may also wonder whether the difference between small and large sign languages is only driven by community size or also by other structural properties such as the fact that all large sign languages are deaf community sign languages, whereas the set of small sign languages included both deaf community sign languages and village sign languages. Deaf community sign languages and village sign languages might differ in the homogeneity of the language users or the density of the community (whether one's contacts are also in contact with each other). These factors have been argued to influence linguistic structure. Interestingly, the sign language that received the lowest iconicity ratings in our study was Estonian Sign Language, which is a deaf community sign language. The ratings for the signs from the other small deaf community sign language (Icelandic Sign Language) rank roughly in the middle of the ratings for the small sign languages—higher than for Estonian Sign Language, Adamorobe Sign Language, and Miyakubo, but lower than the ratings for Kufr Qasem Sign Language and Kata Kolok. Therefore, there does not seem to be a systematic difference in iconicity ratings between these two types of languages, and the effect of community size does not seem to be due to differences in language type.

To summarize, our study demonstrated that the form that signs take is influenced by the size of the community of users, such that sign languages with more users avoid signs that are particularly low in iconicity. The study thus aligns with prior research about sound symbolism in spoken languages which found that languages with larger communities of speakers have more sound symbolic words (Lev‐Ari et al., 2021). The extension of the study to sign languages is particularly interesting as it has been argued that sign languages shed their iconicity with time, and larger sign languages are more likely to be older. The study also opens up new questions. Future research should examine why and how the motivation for iconic forms might differ across semantic domains and how the type of iconicity that signs embody might differ for concrete and abstract concepts.

## Notes

1 If the larger language communities have more developed press, communication systems, or social media platforms, then these could help reduce variability by increasing homogenization. Furthermore, very small and close‐knit language communities, in which all language users are familiar with each other and share cultural knowledge, often show great lexical variation (Meir, Israel, Sandler, Padden, & Aronoff, 2012; Mudd, de Vos, & de Boer, 2022). In these latter cases, there is less pressure to align because past experience with the interlocutor and/or the ability to recover the meaning from the culturally shared iconicity in the form allows successful communication even without convergence on a shared form.

2 One of these participants reported being a native speaker of English rather than German.

3 For a discussion problematizing such dichotic terms, see Hou and de Vos (2022).

4 Initialized signs are signs that incorporate the handshape associated with the first letter of the written word associated with the concept.

5 Fingerspelling involves the production of certain handshapes which are used to represent letters of the written alphabet. As fingerspellings do not represent the concept but letters in the writing system of the spoken language in the region, they were excluded.

6 All reported results hold if the analyses are carried out with *learn* classified as a social concept rather than a nonsocial concept.

7 The algorithms in the package create a vector for each word based on its co‐occurrence with other words. If two words tend to occur in similar contexts, the cosine similarity between their vectors will be high.

8 Language age estimates are from Abner et al. (2024; Supplementary Materials) for American SL, Chinese SL, Estonian SL, and Russian SL; from Miles (2001) for Indian SL; from Bergman and Engberg‐Pedersen (2010) for Icelandic SL; from the establishment of the first school for the Deaf (1857) for LIBRAS, and from De Vos and Nyst (2018) in combination with the authors’ knowledge of the community for Katak Kolok, Miyakubo, Adamarobe SL, and Kufr Qassem SL.

9 At first glance, it might seem that Mudd et al. (2022) model shows the opposite of our study, as their model predicts that larger communities will have fewer iconic signs. Their model, however, focuses on culturally specific iconicity. We also predict that larger communities will be less likely to use culturally specific iconicity as this would hinder communication. In contrast, we focus on iconicity that does not depend on cultural knowledge and can be understood even by outgroup members (e.g., our sign‐naïve participants who come from different cultures than those of the language users). This type of iconicity was not examined in Mudd et al. (2022).

## References

[cogs70074-bib-0001] Abner, N. , Clarté, G. , Geraci, C. , Ryder, R. J. , Mertz, J. , Salgat, A. , & Yu, S. (2024). Computational phylogenetics reveal histories of sign languages. Science, 383(6682), 519–523 38301028 10.1126/science.add7766

[cogs70074-bib-0002] Baus, C. , Carreiras, M. , & Emmorey, K. (2013). When does iconicity in sign language matter? Language and Cognitive Processes, 28(3), 261–271. 23543899 10.1080/01690965.2011.620374 PMC3608132

[cogs70074-bib-0003] Bates , D. M. , Maechler, M. , Bolker, B. , & Walker, S. (2016). lme4: Mixed‐effects modeling with R; 2010. Retrieved from https://cran.r‐project.org/web/packages/lme4

[cogs70074-bib-0004] Bellugi, U. , & Klima, E. (1976). Two faces of sign: Iconic and abstract. Annals of the New York Academy of Sciences, 280(1), 514–538. 1070935 10.1111/j.1749-6632.1976.tb25514.x

[cogs70074-bib-0005] Bergman , B. , & Engberg‐Pedersen, E. (2010). Transmission of sign languages in the Nordic countries. In D. Brentari (Ed.), Sign languages (pp. 74–94). Cambridge University Press.

[cogs70074-bib-0006] Blasi, D. E. , Wichmann, S. , Hammarström, H. , Stadler, P. F. , & Christiansen, M. H. (2016). Sound–meaning association biases evidenced across thousands of languages. Proceedings of the National Academy of Sciences, 11339, 10818–10823. 10.1073/pnas.1605782113 PMC5047153 27621455

[cogs70074-bib-0007] Brand, J. , Monaghan, P. , & Walker, P. (2018). The changing role of sound‐symbolism for small versus large vocabularies. Cognitive Science, 42, 578–590. 29235140 10.1111/cogs.12565 PMC6001752

[cogs70074-bib-0008] Brysbaert, M. , & New, B. (2009). Moving beyond Kucera and Francis: A critical evaluation of current word frequency norms and the introduction of a new and improved word frequency measure for American English. Behavior Research Methods, 41, 977–990. 19897807 10.3758/BRM.41.4.977

[cogs70074-bib-0009] Brysbaert, M. , Warriner, A. B. , & Kuperman, V. (2014). Concreteness ratings for 40 thousand generally known English word lemmas. Behavior Research Methods, 46, 904–911. 24142837 10.3758/s13428-013-0403-5

[cogs70074-bib-0010] Campbell, R. , Martin, P. , & White, T. (1992). Forced choice recognition of sign in novice learners of British Sign Language. Applied Linguistics, 13(2), 185–201.

[cogs70074-bib-0011] Caselli, N. K. , & Pyers, J. E. (2020). Degree and not type of iconicity affects sign language vocabulary acquisition. Journal of Experimental Psychology: Learning, Memory, and Cognition, 46(1), 127–139. 31094562 10.1037/xlm0000713 PMC6858483

[cogs70074-bib-0012] Caselli, N. , Sevcikova Sehyr, Z. , Cohen‐Goldberg, A. M. , & Emmorey, K. (2017). ASL‐LEX: A lexical database of American Sign Language. Behavior Research Methods, 49(2), 784–801. 27193158 10.3758/s13428-016-0742-0 PMC5116008

[cogs70074-bib-0013] Christensen, R. (2023). ordinal—Regression Models for Ordinal Data . R package version 2023.12‐4. Retrieved from https://CRAN.R‐project.org/package=ordinal

[cogs70074-bib-0014] Chambers, J. K. , & Trudgill, P. (1998). Dialectology. Cambridge: Cambridge University Press.

[cogs70074-bib-0015] Dale, R. , & Lupyan, G. (2012). Understanding the origins of morphological diversity: The linguistic niche hypothesis. Advances in Complex Systems, 15(03n04), 1150017.

[cogs70074-bib-0016] De Vos, C. (2012). Sign‐spatiality in Kata Kolok: How a village sign language in Bali inscribes its signing space *(Doctoral dissertation)* . Radboud University Nijmegen.

[cogs70074-bib-0017] De Vos, C. , & Nyst, V. (2018). The time depth and typology of rural sign languages. Sign Language Studies, 18(4), 477–487.

[cogs70074-bib-0018] Dingemanse, M. , Blasi, D. E. , Lupyan, G. , Christiansen, M. H. , & Monaghan, P. (2015). Arbitrariness, iconicity, and systematicity in language. Trends in Cognitive Sciences, 19(10), 603–615. 26412098 10.1016/j.tics.2015.07.013

[cogs70074-bib-0019] Diveica, V. , Pexman, P. M. , & Binney, R. J. (2023). Quantifying social semantics: An inclusive definition of socialness and ratings for 8388 English words. Behavior Research Methods, 55, 461–473. 35286618 10.3758/s13428-022-01810-x PMC10027635

[cogs70074-bib-0020] Eberhard, D. M. , Simons, G. F. , & Fennig, C. D . (2023). Ethnologue: Languages of the world (26th ed.) Dallas, TX: SIL International.

[cogs70074-bib-0021] Fay, N. , & Ellison, T. M. (2013). The cultural evolution of human communication systems in different sized populations: Usability trumps learnability. PLoS One, 8(8), e71781. 23967243 10.1371/journal.pone.0071781 PMC3744464

[cogs70074-bib-0022] Fay, N. , Garrod, S. , & Roberts, L. (2008). The fitness and functionality of culturally evolved communication systems. Philosophical Transactions of the Royal Society B: Biological Sciences, 363(1509), 3553–3561. 10.1098/rstb.2008.0130 PMC2607338 18799421

[cogs70074-bib-0023] Emmorey, K. (2014). Iconicity as structure mapping. Philosophical Transactions of the Royal Society B: Biological Sciences, 369(1651), 20130301. 10.1098/rstb.2013.0301 PMC4123680 25092669

[cogs70074-bib-0024] Frishberg, N. (1975). Arbitrariness and iconicity: Historical change in American Sign Language. Language, 51(3), 696–719.

[cogs70074-bib-0025] Giannakopoulou, A. , Brown, H. , Clayards, M. , & Wonnacott, E. (2017). High or low? Comparing high and low‐variability phonetic training in adult and child second language learners. PeerJ, 5, e3209. 28584698 10.7717/peerj.3209 PMC5452958

[cogs70074-bib-0026] Gimeno‐Martínez, M. , & Baus, C. (2022). Iconicity in sign language production: Task matters. Neuropsychologia, 167, 108166. 35114219 10.1016/j.neuropsychologia.2022.108166

[cogs70074-bib-0027] Heald, S. L. , & Nusbaum, H. C. (2014). Speech perception as an active cognitive process. Frontiers in Systems Neuroscience, 8, 35. 24672438 10.3389/fnsys.2014.00035 PMC3956139

[cogs70074-bib-0028] Hou, L. , & de Vos, C. (2022). Classifications and typologies: Labeling sign languages and signing communities. Journal of Sociolinguistics, 26(1), 118–125. 10.1111/josl.12490

[cogs70074-bib-0029] Imai, M. , Kita, S. , Nagumo, M. , & Okada, H. (2008). Sound symbolism facilitates early verb learning. Cognition, 109, 54–65. 18835600 10.1016/j.cognition.2008.07.015

[cogs70074-bib-0030] Kantartzis, K. , Imai, M. , & Kita, S. (2011). Japanese sound‐symbolism facilitates word learning in English speaking children. Cognitive Science, 353, 575–586.

[cogs70074-bib-0031] Koplenig, A. (2019). Language structure is influenced by the number of speakers but seemingly not by the proportion of non‐native speakers. Royal Society Open Science, 62, 181274. 10.1098/rsos.181274 PMC6408393 30891265

[cogs70074-bib-0032] Koplenig, A. , & Wolfer, S. (2023). Languages with more speakers tend to be harder to (machine‐) learn. Scientific Reports, 13(1), 18521. 37898699 10.1038/s41598-023-45373-z PMC10613286

[cogs70074-bib-0033] Lev‐Ari, S. (2023). The emergence of word order from a social network perspective. Cognition, 237, 105466. 37116321 10.1016/j.cognition.2023.105466

[cogs70074-bib-0034] Lev‐Ari, S. , Kancheva, I. , Marston, L. , Morris, H. , Swingler, T. , & Zaynudinova, M. (2021). ‘Big’ sounds bigger in more widely‐spoken languages. Cognitive Science, 45(11), e13059. 34758146 10.1111/cogs.13059

[cogs70074-bib-0035] Lieberth, A. K. , & Gamble, M. E. B. (1991). The role of iconicity in sign language learning by hearing adults. Journal of Communication Disorders, 24(2), 89–99. 2066475 10.1016/0021-9924(91)90013-9

[cogs70074-bib-0036] Lockwood, G. , Dingemanse, M. , & Hagoort, P. (2016). Sound‐symbolism boosts novel word learning. Journal of Experimental Psychology: Learning, Memory, and Cognition, 42(8), 1274. 26844577 10.1037/xlm0000235

[cogs70074-bib-0037] Lupyan, G. , & Dale, R. (2010). Language structure is partly determined by social structure. PLoS One, 51, e8559. 10.1371/journal.pone.0008559 PMC2798932 20098492

[cogs70074-bib-0038] Lupyan, G. , & Winter, B. (2018). Language is more abstract than you think, or, why aren't languages more iconic? Philosophical Transactions of the Royal Society B: Biological Sciences, 373(1752), 20170137. 10.1098/rstb.2017.0137 PMC6015821 29915005

[cogs70074-bib-0039] McGarry, M. E. , Mott, M. , Midgley, K. J. , Holcomb, P. J. , & Emmorey, K. (2021). Picture‐naming in American Sign Language: An electrophysiological study of the effects of iconicity and structured alignment. Language, Cognition and Neuroscience, 36(2), 199–210. 33732747 10.1080/23273798.2020.1804601 PMC7959108

[cogs70074-bib-0040] McGarry, M. E. , Midgley, K. J. , Holcomb, P. J. , & Emmorey, K. (2023). How (and why) does iconicity effect lexical access: An electrophysiological study of American sign language. Neuropsychologia, 183, 108516. 36796720 10.1016/j.neuropsychologia.2023.108516 PMC10576952

[cogs70074-bib-0041] Meier, R. P. , & Newport, E. L. (1990). Out of the hands of babes: On a possible sign advantage in language acquisition. Language, 66(1), 1–23.

[cogs70074-bib-0042] Meir, I. , Sandler, W. , Padden, C. , & Aronoff, M. (2010). Emerging sign languages. Oxford Handbook of Deaf Studies, Language, and Education, 2, 267–280.

[cogs70074-bib-0043] Meir, I. , Israel, A. , Sandler, W. , Padden, C. A. , & Aronoff, M. (2012). The influence of community on language structure: Evidence from two young sign languages. Linguistic Variation, 12(2), 247–291.

[cogs70074-bib-0044] Meteyard, L. , Stoppard, E. , Snudden, D. , Cappa, S. F. , & Vigliocco, G. (2015). When semantics aids phonology: A processing advantage for iconic word forms in aphasia. Neuropsychologia, 76, 264–275. 25637775 10.1016/j.neuropsychologia.2015.01.042

[cogs70074-bib-0045] Miles, M. (2001). Signs of development in deaf south & south‐west Asia: Histories, cultural identities, resistance to cultural imperialism. Independent Living Institute.

[cogs70074-bib-0046] Mott, M. , Midgley, K. J. , Holcomb, P. J. , & Emmorey, K. (2020). Cross‐modal translation priming and iconicity effects in deaf signers and hearing learners of American Sign Language. Bilingualism: Language and Cognition, 23, 1032–1044. 33897272 10.1017/S1366728919000889 PMC8061897

[cogs70074-bib-0047] Mudd, K. , de Vos, C. , & de Boer, B. (2022). Shared context facilitates lexical variation in sign language emergence. Languages, 7(1), 31.

[cogs70074-bib-0048] Navarrete, E. , Peressotti, F. , Lerose, L. , & Miozzo, M. (2017). Activation cascading in sign production. Journal of Experimental Psychology: Learning, Memory, and Cognition, 43(2), 302–318. 27656874 10.1037/xlm0000312

[cogs70074-bib-0049] Nielsen, A. , & Rendall, D. (2012). The source and magnitude of sound‐symbolic biases in processing artificial word material and their implications for language learning and transmission. Language and Cognition, 42, 115–125.

[cogs70074-bib-0050] Nyst, V. (2007). A descriptive analysis of Adamorobe sign language (Ghana). Netherlands Graduate School of Linguistics. LOT.

[cogs70074-bib-0051] Occhino, C. , Anible, B. , Wilkinson, E. , & Morford, J. P. (2017). Iconicity is in the eye of the beholder: How language experience affects perceived iconicity. Gesture, 16(1), 100–126.

[cogs70074-bib-0052] Ohala, J. J. , Hinton, L. , & Nichols, J. (1997). Sound symbolism. In Proceedings of the 4th Seoul International Conference on Linguistics [SICOL] (pp. 98–103).

[cogs70074-bib-0054] Ortega, G. (2017). Iconicity and sign lexical acquisition: A review. Frontiers in Psychology, 8, 1280. 28824480 10.3389/fpsyg.2017.01280 PMC5539242

[cogs70074-bib-0055] Ortega, G. , & Morgan, G. (2015a). Input processing at first exposure to a sign language. Second Language Research, 31(4), 443–463.

[cogs70074-bib-0056] Ortega, G. , & Morgan, G. (2015b). Phonological development in hearing learners of a sign language: The influence of phonological parameters, sign complexity, and iconicity. Language Learning, 65(3), 660–688.

[cogs70074-bib-0057] Peña, M. , Mehler, J. , & Nespor, M. (2011). The role of audiovisual processing in early conceptual development. Psychological Science, 22, 1419–1421. 21960249 10.1177/0956797611421791

[cogs70074-bib-0058] Perlman, M. , Little, H. , Thompson, B. , & Thompson, R. L. (2018). Iconicity in signed and spoken vocabulary: A comparison between American Sign Language, British Sign Language, English, and Spanish. Frontiers in Psychology, 9, 1433. 30154747 10.3389/fpsyg.2018.01433 PMC6102584

[cogs70074-bib-0059] Perniss, P. , Thompson, R. L. , & Vigliocco, G. (2010). Iconicity as a general property of language: Evidence from spoken and signed languages. Frontiers in Psychology, 1, 227. 21833282 10.3389/fpsyg.2010.00227 PMC3153832

[cogs70074-bib-0060] Pisoni, D. B. (1993). Long‐term memory in speech perception: Some new findings on talker variability, speaking rate and perceptual learning. Speech Communication, 13, 109–125. 21461185 10.1016/0167-6393(93)90063-q PMC3066018

[cogs70074-bib-0061] Pizzuto, E. , & Volterra, V. (2000). Iconicity and transparency in sign languages: A crosslinguistic cross‐cultural view. In K. Emmorey & H. Lane (Eds.), The signs of language revisited: An anthology to honor Ursula Bellugi and Edward Klima (pp. 261–286). Mahwah, NJ: Lawrence Erlbaum Associates.

[cogs70074-bib-0062] R Core Team . (2020). A language and environment for statistical computing . Vienna, Austria: R Foundation for Statistical Computing. URL Retrieved from https://www.R‐project.org/

[cogs70074-bib-0063] Raviv, L. , Meyer, A. , & Lev‐Ari, S. (2019). Larger communities create more systematic languages. Proceedings of the Royal Society B, 2861907, 20191262. 10.1098/rspb.2019.1262 PMC6661353 31311478

[cogs70074-bib-0064] Reali, F. , Chater, N. , & Christiansen, M. H. (2018). Simpler grammar, larger vocabulary: How population size affects language. Proceedings of the Royal Society B: Biological Sciences, 285(1871), 20172586. 10.1098/rspb.2017.2586 PMC5805949 29367397

[cogs70074-bib-0065] Rehurek, R. , & Sojka, P. (2011). Gensim–python framework for vector space modelling. NLP Centre, Faculty of Informatics, Masaryk University.

[cogs70074-bib-0066] Sadakata, M. , & McQueen, J. M. (2014). Individual aptitude in Mandarin lexical tone perception predicts effectiveness of high‐variability training. Frontiers in Psychology, 5, 1318. 25505434 10.3389/fpsyg.2014.01318 PMC4243698

[cogs70074-bib-0067] Sehyr, Z. S. , & Emmorey, K. (2019). The perceived mapping between form and meaning in American Sign Language depends on linguistic knowledge and task: Evidence from iconicity and transparency judgments. Language and Cognition, 11(2), 208–234. 31798755 10.1017/langcog.2019.18 PMC6886719

[cogs70074-bib-0068] Sevcikova Sehyr, Z. , Caselli, N. , Cohen‐Goldberg, A. M. , & Emmorey, K. (2021). The ASL‐LEX 2.0 Project: A database of lexical and phonological properties for 2,723 signs in American Sign Language. Journal of Deaf Studies and Deaf Education, 26(2), 263–277. 33598676 10.1093/deafed/enaa038 PMC7977685

[cogs70074-bib-0069] Shcherbakova, O. , Michaelis, S. M. , Haynie, H. J. , Passmore, S. , Gast, V. , Gray, R. D. , Greenhill, S. J. , Blasi, D. E. , & Skirgård, H. (2023). Societies of strangers do not speak less complex languages. Science Advances, 9(33), eadf7704 37585533 10.1126/sciadv.adf7704 PMC10431698

[cogs70074-bib-0070] Shinohara, K. , & Kawahara, S. (2010). A cross‐linguistic study of sound symbolism: The images of size. Annual Meeting of the Berkeley Linguistics Society, 36(1), 396–410.

[cogs70074-bib-0071] Sidhu, D. M. , & Pexman, P. M. (2018a). Five mechanisms of sound symbolic association. Psychonomic Bulletin & Review, 255, 1619–1643. 10.3758/s13423-017-1361-1 28840520

[cogs70074-bib-0072] Sidhu, D. M. , & Pexman, P. M. (2018b). Lonely sensational icons: Semantic neighbourhood density, sensory experience and iconicity. Language, Cognition and Neuroscience, 33(1), 25–31.

[cogs70074-bib-0073] Sidhu, D. M. , Vigliocco, G. , & Pexman, P. M. (2020). Effects of iconicity in lexical decision. Language and Cognition, 121, 164–181.

[cogs70074-bib-0074] Stamp, R. , Omar‐Hajdawood, D. , & Novogrodsky, R. (2024). Topical influence: Reiterative code‐switching in the Kufr Qassem Deaf Community. Sign Language Studies, 24(4), 771–802.

[cogs70074-bib-0075] Tamariz, M. , Roberts, S. G. , Martínez, J. I. , & Santiago, J. (2018). The interactive origin of iconicity. Cognitive Science, 42(1), 334–349. 28503811 10.1111/cogs.12497

[cogs70074-bib-0076] Taub, S. F. (2001). Language from the body: Iconicity and metaphor in American Sign Language. Cambridge University Press.

[cogs70074-bib-0077] Thompson, R. L. , Vinson, D. P. , Woll, B. , & Vigliocco, G. (2012). The road to language learning is iconic: Evidence from British Sign Language. Psychological Science, 2312, 1443–1448. 10.1177/0956797612459763 23150275

[cogs70074-bib-0078] Vinson, D. , Thompson, R. L. , Skinner, R. , & Vigliocco, G. (2015). A faster path between meaning and form? Iconicity facilitates sign recognition and production in British Sign Language. Journal of Memory and Language, 82, 56–85.

[cogs70074-bib-0079] Wickham, H. , Averick, M. , Bryan, J. , Chang, W. , D'Agostino McGowan, L. , François, R. , Grolemund, G. , Hayes, A. , Henry, L. , Hester, J. , Kuhn, M. , Pedersen, T. L. , Miller, E. , Bache, S. M. , Müller, K. , Ooms, J. , Robinson, D. , Seidel, D. P. , Spinu, V. , Takahashi, K. , Vaughan, D. , Wilke, C. , Woo, K. , & Yutani, H. (2019). Welcome to the tidyverse. Journal of Open Source Software, 443, 1686.

[cogs70074-bib-0080] Winter, B. , Lupyan, G. , Perry, L. K. , Dingemanse, M. , & Perlman, M. (2024). Iconicity ratings for 14,000+ English words. Behavior Research Methods, 56, 1640–1655. 37081237 10.3758/s13428-023-02112-6

[cogs70074-bib-0081] Wray, A. , & Grace, G. W. (2007). The consequences of talking to strangers: Evolutionary corollaries of socio‐cultural influences on linguistic form. Lingua, 117(3), 543–578.

[cogs70074-bib-0082] Yano, U. , & Matsuoka, K. (2018). Numerals and timelines of a shared sign language in Japan. Sign Language Studies, 18(4), 640–665.

